# An Altered Lipid Profile Is Indicative of Increased Insulin Requirement in Children and Adolescents at the Onset of Type 1 Diabetes

**DOI:** 10.3390/jpm16010014

**Published:** 2026-01-01

**Authors:** Giulio Maltoni, Luca Bernardini, Andrea Scozzarella, Giulia Montanari, Erika Cantarelli, Marcello Lanari

**Affiliations:** 1 Pediatric Unit, IRCCS Azienda Ospedaliero-Universitaria di Bologna, 40138 Bologna, Italy; giulio.maltoni@aosp.bo.it (G.M.); andrea.scozzarella@aosp.bo.it (A.S.); marcello.lanari@unibo.it (M.L.); 2 Department of Medical and Surgical Sciences, Alma Mater Studiorum—University of Bologna, Via Massarenti 9, 40138 Bologna, Italy; 3 Specialty School of Pediatrics, Alma Mater Studiorum—University of Bologna, 40138 Bologna, Italy; giulia.montanari22@studio.unibo.it; 4 Unit of Pediatrics, University Hospital of Parma, 43126 Parma, Italy; erika.cantarelli@ao.pr.it

**Keywords:** Type 1 Diabetes, lipid profile, insulin requirement, ketoacidosis, triglycerides

## Abstract

**Background**: At the onset of Type 1 Diabetes (T1D), international guidelines recommend initiating subcutaneous insulin therapy within a wide dosage range (0.5–1 IU/kg/day), as insulin requirement (IR) varies greatly based on several factors, including age, pubertal status, and the presence of diabetic ketoacidosis (DKA). In clinical practice, some individuals require higher-than-expected IR, leading to prolonged hospitalization. This study aimed to identify predictive factors for elevated IR at T1D onset. **Methods**: We conducted a retrospective observational study including 218 children and adolescents diagnosed with T1D between January 2010 and September 2020. Clinical and laboratory parameters were collected. IR was defined as the highest daily subcutaneous insulin dose (IU/kg/day) during hospitalization, after resolution of DKA. **Results**: As expected, DKA severity and HbA1c levels were associated with increased IR. However, the strongest independent predictor in the multivariate model was serum triglyceride level (β = 0.27, *p* < 0.001), with an adjusted R^2^ of 0.37. No evidence of multicollinearity was detected, and ROC analysis yielded an AUC of approximately 0.70. **Conclusions**: Hypertriglyceridemia at T1D onset is independently associated with higher IR, regardless of DKA severity. Early recognition of this marker could help optimize insulin dosing, improve metabolic stabilization, and potentially shorten hospital stays.

## 1. Introduction

Type 1 Diabetes Mellitus (T1D) is an autoimmune disorder characterized by progressive pancreatic β-cell destruction, leading to insulin deficiency and chronic hyperglycemia. Clinical presentation is heterogeneous and is influenced by diagnostic delay [1]. Typical symptoms include polyuria, polydipsia, nocturia, enuresis, weight loss, polyphagia, and fatigue. In delayed cases, diabetic ketoacidosis (DKA) may occur due to severe insulin deficiency and counter-regulatory hormone activation. DKA is characterized by dehydration, tachypnea, acetonemic breath, vomiting, abdominal pain, altered consciousness, and coma [1,2]. T1D is a major public health issue, both due to the increasing incidence observed in most populations in recent decades, with an annual growth rate of 3–4% [3], and due to the challenges of disease management, caused by the risk of acute and chronic complications [4,5]. Insulin therapy is essential to restore normal cellular metabolism, to suppress lipolysis and ketogenesis, and to normalize blood glucose concentrations [1,2]. National and international guidelines for the management of diabetic ketoacidosis include, among other procedures, intravenous (IV) administration of insulin at a dosage ranging from 0.05 to 0.1 IU/kg/h [1,2,6]. Once the ketoacidosis has resolved, it is possible to switch to an insulin regimen involving multiple-daily subcutaneous administration. Children and adolescents with T1D onset should be managed in experienced centers in the management of this condition, generally through an inpatient stay lasting several days, in which good metabolic control is achieved, and the family is provided with all the information on the complex home management of the disease. The recommended doses of starting subcutaneous insulin therapy are quite variable and are able to range from 0.5 to 1 IU/kg/day. The range is wide because there are many factors that can influence individual insulin sensitivity, such as age [6,7], gender [7,8], pubertal stage, thyroid function [9], and degree of ketoacidosis. However, frequently, many children and/or adolescents at the diagnosis of T1D may require a higher daily dose of insulin than expected, resulting in the need for a longer time to reach stable blood glucose values and, consequently, more hospitalization days. In this context, insulin requirement (IR) refers to the total daily subcutaneous insulin dose per kilogram of body weight required to achieve metabolic stabilization during the early phase of the disease and serves as a practical clinical indicator of reduced insulin sensitivity at diagnosis. Beyond glycemic indices and acid–base status, lipid metabolism is profoundly altered at T1D onset. Absolute insulin deficiency and the surge of counter-regulatory hormones promote adipose lipolysis and hepatic very-low-density lipoprotein (VLDL) production, while reduced lipoprotein lipase activity limits triglyceride clearance. This results in transient hypertriglyceridemia, a plausible biochemical indicator of acute metabolic stress and reduced insulin sensitivity. Although guidelines provide detailed recommendations for DKA management and initial insulin dosing ranges [1,2,6], few studies have explored whether routine lipid parameters at admission offer prognostic value regarding early IR in children and adolescents with new-onset T1D. Clarifying this relationship is clinically relevant, given the wide inter-individual variability in IR at diagnosis and the potential consequences for length of stay, resource use, and early glycemic stabilization. We therefore investigated whether lipid profile—particularly serum triglycerides—measured at presentation is associated with higher IR independently of metabolic derangement (e.g., DKA severity) and other baseline factors, aiming to provide a simple, readily available marker to support individualized insulin titration at onset.

The main goal of our study was to identify predictors of reduced insulin sensitivity in children and adolescents at T1D onset in order to help physicians to initiate appropriate therapy more quickly by achieving the correct therapeutic dosage, thereby obtaining good metabolic compensation early on and consequently reducing hospital stay and costs. Secondarily, we aimed to evaluate IR at least 1 year after T1D onset to check the influence of factors leading to reduced insulin sensitivity after the disease’s diagnosis.

## 2. Materials and Methods

### 2.1. Objective of the Study

The primary objective of the study was to assess the presence of predictive factors (clinical and/or laboratory) at T1D onset related to a higher IR in a population of children and adolescents diagnosed with T1D. The secondary objective of the study was to evaluate IR at least 1 year after T1D onset.

### 2.2. Study Cohort and Design

This was a retrospective, observational, monocentric, non-pharmacological study that included children and adolescents aged 1–18 years consecutively admitted to the Pediatric Endocrinology Unit of the IRCCS AOU S. Orsola in Bologna between 1 January 2010 and 21 September 2020 with new-onset T1D. Patients with acute infection, corticosteroid use, hepatic or renal dysfunction, or incomplete 12-month follow-up were excluded. All participants had positive pancreatic autoantibodies confirming autoimmune etiology. Cases with missing baseline data represented <5% of the initial dataset and were excluded to ensure analytical consistency.

Patients were treated according to the International Society for Pediatric and Adolescent Diabetes (ISPAD) guidelines [4,6]. Insulin therapy was initiated intravenously (0.05–0.1 IU/kg/h) during ketoacidosis and then transitioned to a multidose subcutaneous regimen upon DKA resolution. The initial Total Daily insulin Dose (TDD) was calculated according to body weight and corrected depending on blood-glucose levels. Insulin requirement (IR) was defined as the highest total daily subcutaneous insulin dose (IU/kg/day) administered after resolution of diabetic ketoacidosis and before hospital discharge. This methodological choice was intentional to reflect acute insulin resistance at disease onset rather than stabilized insulin requirements after metabolic compensation. Subjects were discharged when they had completed diabetes education and achieved adequate glycemic control [10,11]. Data were collected retrospectively through electronic medical records (Metaclinic^®^) and in-patient medical records. Admissions were identified through pediatric emergency department registry and informed consent was obtained from parents.

The following data were collected for all enrolled subjects:

1. Clinical parameters at diagnosis: Sex, age, body weight (kg), height (cm), body mass index (BMI), degree of weight loss at diagnosis (in % of reported weight), duration of diabetes symptoms before diagnosis, pubertal stage, presence of celiac disease or autoimmune thyroiditis, any other medical treatment. Pubertal status was assessed according to Tanner staging and included as a covariate in the analyses. Standard Deviations (SDs) for height, weight, and BMI were adjusted for age and gender according to Italian reference standards [12]. Celiac disease was diagnosed according to the ESPGHAN guidelines in force at the time of diagnosis, based on positive serological testing, with histological confirmation by duodenal biopsy when indicated [13,14].
2. Laboratory parameters at diagnosis: Blood glucose (mg/dL), HbA1c (% and mmol/mol), fructosamine (μmol/L), presence of DKA (defined by venous pH < 7.3 and/or bicarbonate (HCO_3_^−^) < 15 mmol/L) [15], C-peptide (ng/mL), total cholesterol (mg/dL), HDL-cholesterol (mg/dL), triglycerides (mg/dL), uric acid (mg/dL), thyroid-stimulating hormone (TSH, microU/mL), and free thyroxine (fT4, pg/mL). Lipid profile was measured on the first blood sample collected at admission, before the initiation of intravenous insulin and, whenever feasible, before glucose infusion and extended rehydration. Although a formal fasting state could not be ensured, most patients—particularly those presenting with DKA—had markedly reduced oral intake for several hours before admission, resulting in a functional fasting condition. Therefore, triglyceride concentrations plausibly reflected a fasting-like metabolic condition and the acute metabolic stress at presentation, including lipolysis associated with DKA. Serum C-peptide levels were measured at diagnosis as a marker of residual β-cell function and were included among the covariates in both univariate and multivariable analyses assessing determinants of IR.
3. Insulin therapy regimen during hospitalization: During hospitalization, insulin therapy was initiated and titrated according to the ISPAD 2022 guidelines for new-onset T1D. Glycemic targets were maintained between 100 and 180 mg/dL, with gradual adjustment of basal and bolus components under daily supervision by at least two pediatric endocrinologists, ensuring consistency and minimizing inter-observer variability. As defined above, IR was the highest total daily subcutaneous insulin dose (IU/kg/day) after DKA resolution and prior to discharge, reflecting the stable metabolic phase following rehydration. The highest subcutaneous insulin dose was chosen to capture the phase of maximal insulin resistance during acute metabolic instability, whereas discharge doses may underestimate early insulin needs due to subsequent titration aimed at hypoglycemia prevention. Subcutaneous TDD was recorded daily and expressed in IU/day and IU/kg, adjusted to the weight measured at admission. IV regimen was time-limited and restricted to patients with DKA at onset, according to ISPAD guidelines [1,6]. IR (IU/kg/day) was calculated by dividing TDD per body weight at diagnosis.
4. Clinical parameters at discharge: Intravenous hydration duration (hours) and hospitalization duration (days).
5. Clinical and laboratory parameters at 12 months’ follow-up: HbA1c (% and mmol/mol), total cholesterol (mg/dL), HDL-cholesterol (mg/dL), pubertal stage, and IR (IU/kg/day). The 12-month follow-up was selected as the secondary time-point because it generally represents the end of the partial remission (honeymoon) phase and the stabilization of insulin therapy, according to ISPAD 2022 guidelines [6]. A shorter (6-month) follow-up would still reflect honeymoon variability, while a longer (24-month) period would reduce the number of evaluable subjects and introduce heterogeneity due to pubertal changes.

### 2.3. Statistical Analysis

All statistical analyses were performed using Jasp 0.17.2.1. To characterize the cohort, descriptive statistics for discrete (frequencies and percentages) and continuous (mean, standard deviation, median and range or interquartile range, IQR) variables were computed for demographics, clinical characteristics, and laboratory measurements. All baseline variables from the first day of admission were considered independent variables in a multivariable linear regression model applying a backward stepwise approach with a nominal type I error of 5%. Multicollinearity was tested (variance inflation factor < 3)**.** Student’s *t*-test was used in the case of a normal distribution of variables, or the non-parametric Mann–Whitney test in the other case. Correlations between clinical and biochemical parameters were assessed by Spearman’s or Pearson’s correlation tests. Model fit was evaluated using the adjusted R^2^, and the predictive ability was explored through a ROC curve analysis (area under the curve, AUC). All results with p equal to or less than 0.05 were considered significant. Missing data were handled by complete-case analysis. Patients without follow-up data were excluded from longitudinal analyses.

### 2.4. Ethics Approval

The study protocol was approved by the local institutional ethics committee (ESORD1T Internal code 323/2020 OSS/AOUBO) and conducted in accordance with the Declaration of Helsinki.

## 3. Results

### 3.1. Cohort Description

The cohort included 218 individuals with new-onset T1D (Table 1); 45.4% were female. Mean age was 8.2 years (±4.1). DKA was diagnosed in 81 patients (37.2%), while severe DKA (pH < 7.10) was present in 31 subjects (14.2%). Mean HbA1c was 101.0 mmol/mol (±24.5). The mean reported weight loss at admission was 11.3% (±7.1) and the mean duration of symptoms was 26.2 days (±23.9); 62.4% of the patients had been symptomatic for at least two weeks. Only eight subjects (3.7%) were asymptomatic and their T1D diagnosis was due to routine examinations or for family history for diabetes. The mean duration of intravenous hydration was 22.0 h (±32.4), while the mean duration of hospitalization was 9.4 days (±3.2). Mean total cholesterol was 185 mg/dL (±61, IQR 67.0), HDL-cholesterol was 50 mg/dL (±31, IQR 22.0), and triglycerides were 179 mg/dL (±156, IQR 137.0). C-peptide at onset was 0.55 ng/mL (±0.40). The mean IR during hospitalization was 0.92 IU/kg/day (±0.39), and all subjects were under a Multiple Daily Injection (MDI) regimen at discharge. Celiac disease was present in 12.8% of individuals, while autoimmune thyroiditis in 14.7%.

### 3.2. Subgroups Description

Dividing subjects on the basis of IR (Table 1), with a cut-off of 1 IU/kg/day (the upper limit according to the ISPAD guidelines), HbA1c was significantly lower in patients with lower IR (97.1 ± 24.2 versus 108.8 ± 21.8), as were admission blood glucose (413 ± 157 versus 529 ± 285), fructosamine (550.1 ± 161.8 versus 590.7 ± 158.9), triglycerides (153 ± 137 versus 233 ± 180), duration of hospitalization (8.8 ± 2.7 versus 10.4 ± 3.6), and duration of hydration (17.3 ± 20.3 versus 28.9 ± 42.9). In patients with higher IR, HDL-cholesterol was significantly lower (46.9 ± 36.8 versus 51.9 ± 29.6), as were mean pH (7.20 ± 0.16 versus 7.29 ± 0.16) and bicarbonate (14.2 ± 8.3 versus 19.1 ± 7.4), with a higher incidence of DKA (54.1% versus 29.2%) and severe DKA (25.7% versus 8.5%). C-peptide was significantly higher in individuals with lower IR. On the other hand, no statistically significant differences emerged regarding mean age at diagnosis, total cholesterol, thyroid function, duration of symptoms, pubertal stage, presence of celiac disease, autoimmune thyroiditis, as well as the presence of first-degree relatives (FDRs) with T1D. Differences in absolute body weight between groups reflect age differences, whereas body size comparisons and regression analyses were based on BMI expressed as age- and sex-adjusted standard deviation scores. No significant correlation was found between triglycerides and BMI at diagnosis; although a mild positive trend was observed, with higher TG values in patients with greater BMI.

As a statistically significant difference in triglycerides was found, we divided the subjects according to triglyceride values at T1D onset (Table 2), with a laboratory cut-off value of 150 mg/dL. This analysis showed that the rates of DKA and severe DKA were significantly higher in patients with hypertriglyceridemia (55.6% versus 24.8% and 21.0% versus 7.1%, respectively). Furthermore, there was a significant difference in terms of admission blood glucose (513.7 ± 268.0 mg/dL versus 408.1 ± 163.8 mg/dL), fructosamine (649.9 ± 160.6 versus 506.4 ± 142.7), HbA1c 108.0 ± 24.6 mmol/mol versus 96.5 ± 22.8 mmol/mol), total cholesterol (210.9 ± 73.3 versus 166.3 ± 42.3), HDL-cholesterol (44.9 ± 35.3 versus 54.3 ± 28.4), and IR (1.06 ± 0.37 versus 0.80 ± 0.34). There was also a significant difference concerning thyroid function (higher TSH with a lower fT4 value) between the two groups. In children and adolescents with hypertriglyceridemia, duration of symptoms, duration of hydration, and duration of hospitalization were significantly higher. There were no statistically significant differences concerning mean age, body weight, weight loss and C-peptide. We subsequently considered only subjects with DKA onset and divided them based on triglycerides, always using a cut-off value of 150 mg/dL, but no statistically significant difference emerged in terms of IR; the same finding also emerged when considering only subjects with severe DKA.

When analyzing differences according to gender, the only significant differences emerged in terms of total cholesterol and C-peptide at onset, with higher values in females (193.3 ± 53.1 versus 178.0 ± 66.2 [*p* < 0.004] and 0.60 ± 0.43 versus 0.51 ± 0.36 [*p* < 0.04], respectively).

Considering the pubertal stage at onset, the only difference found between the prepubertal and pubertal stages was related to C-peptide values (0.43 ± 0.48 versus 0.55 ± 0.38; *p* < 0.03), while IR, although higher in the pubertal stage, did not reach statistical significance. We further described IR variability at T1D onset using subgroups based on baseline characteristics present at diagnosis, including age, gender, BMI, symptom duration, presence or absence of weight loss, and DKA and lipid profile. A multivariable analysis was performed to assess the impact of these characteristics on IR and the most significant variable at multiple regression was triglycerides (*p* < 0.001).

**Figure 1:**
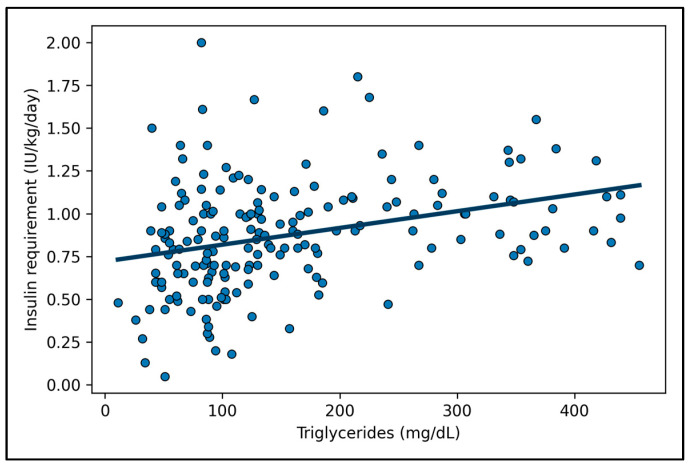
Scatter plot showing the association between serum triglyceride levels at diagnosis and IR (IU/kg/day) during hospitalization. Each dot represents an individual patient. The solid line indicates the linear regression fit. A positive association was observed between triglyceride levels and IR (Pearson r = 0.32, *p* < 0.001).

**Figure 2:**
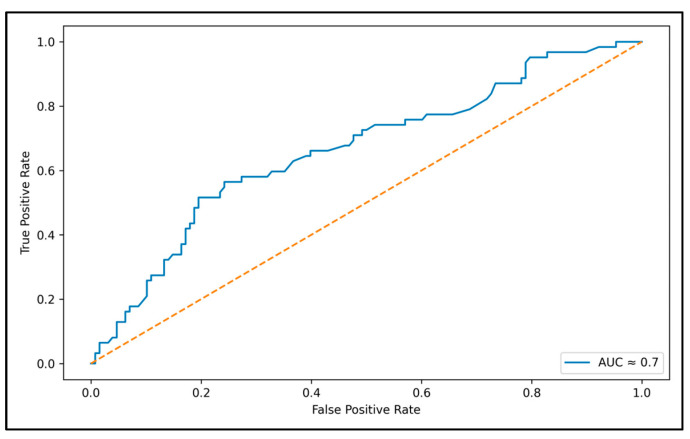
Receiver operating characteristic (ROC) curve assessing the ability of serum triglyceride levels at diagnosis to discriminate patients with higher IR (>1 IU/kg/day). The area under the curve (AUC ≈ 0.70) indicates acceptable discriminative performance, supporting triglycerides as a contributory marker of increased IR at T1D onset.

Although C-peptide was significantly associated with IR in univariate analyses, in the multivariable linear regression model, serum triglyceride level remained the strongest independent predictor of higher IR, after adjustment for DKA severity, HbA1c, BMI z-score and C-peptide (Table 3). The association was moderate but consistent (standardized β = 0.27, *p* < 0.001), and the model explained approximately 34–38% of the variance in IR (adjusted R^2^ ≈ 0.37). Variance inflation factor (VIF) values were below 3, excluding multicollinearity among covariates. A post hoc ROC analysis showed an AUC close to 0.70, supporting acceptable predictive accuracy for clinical interpretation. This level of discrimination indicates an acceptable, non-perfect discriminative performance. The association between triglyceride levels and IR is illustrated in Figure 1, while the discriminative ability of triglycerides for higher IR is shown in Figure 2.

Considering the 12-month follow-up according to IR at T1D onset (Table 4), no statistically significant differences emerged regarding HbA1c, total cholesterol, HDL-cholesterol and C-peptide, except for IR at 12 months, which was still lower in patients with lower IR at T1D onset.

Considering the 12-month follow-up and dividing subjects according to triglycerides at T1D onset, no significant difference emerged regarding the parameters considered. The presence or absence of DKA at T1D onset had a statistically significant correlation with IR at 12 months, with lower IR in patients without DKA (0.60 ± 0.34 versus 0.68 ± 0.25, *p* < 0.015).

## 4. Discussion

In children and adolescents with new-onset T1D, consensus guidelines on initial insulin dosing remain rather non-specific, allowing for wide individual variation in clinical decision-making. The wide range of initial insulin dose and the consequent need for adjustment can prolong hospitalization time, costs and glycemic variability. To the best of our knowledge, our study is the first to specifically focus on factors that can affect IR other than age and metabolic derangement parameters.

As expected, the percentage of weight loss at admission is an important predictor of IR because it is an expression of the acute metabolic dysregulation that occurs at T1D onset, a result also found in another study [16].

Gender was not associated with IR in our cohort, in contrast to previous studies reporting higher insulin resistance in females [7,8,17]. However, such differences are often influenced by age, pubertal stage, and visceral fat distribution. While age and pubertal stage were accounted for in our analysis, visceral adiposity was not specifically assessed and may represent a potential confounder. Interestingly, females showed higher C-peptide and total cholesterol levels at onset, consistent with known sex-related physiological differences [18,19].

Although pubertal stage is known to affect insulin sensitivity due to physiological hormonal changes occurring during adolescence, in our cohort, it did not significantly correlate with IR and was therefore excluded from the final regression analysis. This aligns with previous evidence showing that pubertal insulin resistance is variable and context-dependent in pediatric populations [20].

The weak and non-significant association between triglycerides and BMI observed in our cohort suggests that the transient hypertriglyceridemia at T1D onset is mainly related to acute metabolic stress rather than to pre-existing adiposity. Taken together, the lack of association between triglycerides and BMI at diagnosis, along with the normalization of triglyceride levels at 12-month follow-up, supports the interpretation of hypertriglyceridemia as a transient marker of acute catabolic insulin resistance rather than chronic obesity-related dyslipidemia. During the catabolic phase, increased counter-regulatory hormone activity promotes lipolysis and hepatic VLDL production, while reduced lipoprotein lipase activity impairs TG clearance. Consequently, TG elevations at diagnosis likely reflect acute insulin resistance rather than chronic overweight. This interpretation aligns with previous evidence describing stress-induced dyslipidemia as a metabolic hallmark of the early decompensated phase of T1D [21]. Importantly, the independent association between triglyceride levels and IR persisted after accounting for residual endogenous insulin secretion, as assessed by C-peptide, suggesting that hypertriglyceridemia reflects IR beyond differences in β-cell reserve at diagnosis.

The presence of DKA at onset has a significant impact on HbA1c, fructosamine, glucose, lipid profile (total cholesterol and triglycerides), free thyroid hormone fraction, hydration, hospitalization duration, and residual pancreatic function, expressed as C-peptide values. Previous studies showed that patients with worse metabolic status at presentation required more insulin [22], as well as showing a strong correlation between IR and ketone concentration, which strongly correlates with venous pH and bicarbonate [23]. The negative impact of DKA at T1D onset on metabolic control was also reflected in the long term; in fact, subjects who had DKA at T1D onset seemed to have higher HbA1c values one year later, showing higher IR. Metabolic status at T1D onset strongly influences IR. Venous pH and serum bicarbonate levels at admission—markers of DKA severity—showed a significant correlation with IR. This relationship is expected, considering the pathophysiology of T1D and the presence of insulin resistance at onset, which progressively increases with metabolic dysregulation, as also highlighted by Beckers et al. [16]. These findings provide a biologically plausible explanation for the predictive role of triglycerides at disease onset. The observed relationship between elevated triglyceride levels and higher IR likely reflects a transient state of insulin resistance induced by acute catabolic stress. During DKA, counter-regulatory hormones such as catecholamines, cortisol, and growth hormone stimulate adipose lipolysis and enhance hepatic VLDL synthesis, while reduced lipoprotein lipase activity impairs triglyceride clearance. These combined mechanisms result in stress-induced hypertriglyceridemia, a biochemical marker of diminished insulin sensitivity during the early catabolic phase of T1D. In our multivariable regression model, hypertriglyceridemia emerged as the strongest independent predictor of IR (*p* < 0.001), underscoring its clinical relevance as an early, objective indicator of metabolic severity. Our data suggest that triglyceride levels, as a marker of altered lipid profile, could be integrated—alongside DKA severity, weight, and pubertal stage—into clinical algorithms for estimating initial insulin needs. The typical normalization of triglycerides within one year further supports their interpretation as a marker of acute metabolic decompensation rather than underlying dyslipidemia. The 12-month time-point was intentionally selected to capture a stable metabolic phase after the end of partial remission and before major pubertal influences on insulin sensitivity occur. This choice ensured greater homogeneity and reduced confounding related to residual β-cell function or treatment adjustments. Although our findings require validation in larger multicenter cohorts, they suggest that integrating lipid parameters into early management algorithms could offer a practical and inexpensive approach to support personalized insulin therapy in pediatric T1D.

When analyses were restricted to patients presenting with DKA or severe DKA, no significant difference in IR emerged according to triglyceride levels. This finding may reflect the limited number of subjects within these subgroups, which reduces the statistical power and prevents firm conclusions from being drawn. Larger multicenter cohorts are needed to verify whether the same trend persists in DKA-only populations.

The presence of significant differences in thyroid function in subjects with hypertriglyceridemia is mainly due to the influence of metabolic derangement. Similarly, it is always consequent to the pathophysiology of T1D to have found significantly lower C-peptide values in prepubertal children who, on average, more frequently have DKA at T1D onset and, consequently, have a lower pancreatic reserve.

This study has several strengths, such as the study design, aimed to identify factors present at T1D onset that are potentially related to the IR. Moreover, the potential implication of our study is double: starting insulin treatment at an appropriate dosage allows for achieving an adequate metabolic control more quickly and, thus, for reducing the duration and costs of hospitalization. However, this study has several limitations. First, its retrospective and monocentric design restricts the generalizability of the findings, as patient characteristics and treatment protocols may differ across centers and ethnic groups. Second, although formal fasting conditions were not systematically ensured, triglyceride measurements were obtained prior to insulin initiation and likely reflect acute metabolic stress at presentation rather than underlying chronic dyslipidemia. Third, although insulin titration followed standardized ISPAD-based protocols and reviewed by at least two pediatric endocrinologists, some degree of clinical judgment variability cannot be completely excluded. Fourth, the analysis did not account for all potential confounders—such as hydration status or subclinical infections—which could transiently influence triglyceride levels or insulin sensitivity. Patients with acute infection or corticosteroid exposure were excluded whenever documented, but unrecognized cases cannot be ruled out. Fifth, while C-peptide was analyzed as a continuous variable, autoantibody data were available only as categorical (positive/negative), limiting the ability to fully account for residual β-cell function or immunological heterogeneity. Finally, the sample size was relatively small and follow-up limited to one year. Although the 12-month time-point was chosen to capture the end of the partial remission phase, a longer observation period would be valuable to assess the persistence of these associations and their implications for long-term metabolic outcomes. Prospective multicenter studies with extended follow-up are warranted to validate these findings and clarify their predictive utility in clinical practice. Although higher admission triglycerides were associated with greater IR, translating this relationship into a practical titration protocol remains challenging due to the complex dynamics of insulin sensitivity and metabolic recovery during the first days of therapy. Future studies should evaluate whether incorporating triglyceride assessment into insulin titration algorithms can effectively shorten time to metabolic stabilization and hospital stay.

## 5. Conclusions

Hypertriglyceridemia at T1D onset, reflecting acute metabolic decompensation and transient insulin resistance, is independently associated with higher IR, regardless of DKA severity or BMI. These findings suggest that early lipid alterations mirror transient insulin resistance during metabolic stress and could serve as a simple, readily available marker to anticipate individual insulin needs at diagnosis.

Beyond their immediate clinical relevance, these results highlight the role of lipid metabolism as an integral component of the pathophysiology of new-onset T1D. From a practical standpoint, incorporating triglyceride assessment into initial management may help refine early insulin titration and support a more personalized therapeutic approach.

Future multicenter prospective studies with extended follow-up are warranted to confirm these findings, clarify underlying mechanisms linking lipid metabolism and insulin sensitivity at disease onset, and establish evidence-based thresholds for clinical application. Integrating triglyceride dynamics with complementary metabolic and immunological markers—such as C-peptide, continuous glucose monitoring (CGM) metrics, and autoantibody profiles—could ultimately lead to predictive models that guide individualized therapy from the earliest stages of T1D.

## Figures and Tables

**Table 1:** Demographic, clinical, and laboratory characteristics at T1D onset and comparison of different IRs.

	All	IR ≤ 1 IU/kg/Day	IR > 1 IU/kg/Day	*p*-Value
**Mean age (years)**	8.2 ± 4.1	8.8 ± 4.0	7.6 ± 4.1	NS
**Body weight (kg)**	29.8 ± 15.5	32.4 ± 16.1	24.5 ± 12.5	0.001
**Weight loss (%)**	11.3 ± 7.1	9.6 ± 6.0	13.8 ± 8.5	0.01
**BMI z-score**	−0.71 ± 1.25	−0.60 ± 1.29	−0.90 ± 1.22	NS
**DKA (%)**	37.2%	29.2%	54.1%	0.02
**Severe DKA (%)**	14.2%	8.5%	25.7%	0.009
**HbA1c (mmol/mol)**	101.0 ± 24.5	97.1 ± 24.2	108.8 ± 21.8	<0.001
**Admission blood glucose (mg/dL)**	451.5 ± 216.0	413.2 ± 156.7	529.4 ± 285.0	<0.001
**C-peptide (ng/mL)**	0.55 ± 0.40	0.63 ± 0.44	0.41 ± 0.25	0.001
**Fructosamine (** **μmol/L)**	565.1 ± 164.5	550.1 ± 161.8	590.7 ± 158.9	0.04
**IR (IU/kg/day)**	0.92 ± 0.39	-	-	-
**Total cholesterol (mg/dL)**	184.8 ± 61.0	179.1 ± 48.7	195.0 ± 81.5	NS
**HDL-cholesterol (mg/dL)**	50.3 ± 31.5	51.9 ± 29.6	46.9 ± 36.8	0.001
**Triglycerides (mg/dL)**	179.1 ± 156.1	153.1 ± 136.9	232.6 ± 179.5	<0.001
**TSH (microU/mL)**	3.6 ± 4.3	4.1 ± 5.4	3.0 ± 1.7	NS
**fT4 (pg/mL)**	9.9 ± 1.8	10.0 ± 1.7	10.3 ± 9.7	NS
**Duration of symptoms (days)**	26.2 ± 23.9	24.7 ± 23.6	25.4 ± 23.5	NS
**Duration of hydration (hours)**	22.0 ± 32.4	17.3 ± 20.3	28.9 ± 42.9	0.002
**Duration of hospitalization (days)**	9.4 ± 3.2	8.8 ± 2.8	10.4 ± 3.6	0.013
**pH**	7.26 ± 0.16	7.29 ± 0.16	7.20 ± 0.16	<0.001
**HCO_3_^−^ (mmol/L)**	17.3 ± 7.9	19.0 ± 7.2	14.2 ± 8.3	<0.001
**Celiac disease**	12.8%	13.3%	13.3%	NS
**Autoimmune thyroiditis**	14.7%	14.8%	16.0%	NS
**FDR**	4.1%	5.2%	2.7%	NS
**Prepubertal stage**	46.8%	46.4%	47.4%	NS

NS: not significant.

**Table 2:** Demographic, clinical, and laboratory characteristics at T1D onset according to triglyceride values.

	TG ≤ 150 mg/dL (T1D Onset)	TG > 150 mg/dL (T1D Onset)	*p*-Value
**Mean age (years)**	8.5 ± 3.7	8.1 ± 4.6	NS
**Body weight (kg)**	31.1 ± 14.9	28.9 ± 16.7	NS
**Weight loss (%)**	9.9 ± 5.9	12.6 ± 7.1	NS
**DKA (%)**	24.8%	55.6%	0.004
**Severe DKA (%)**	7.1%	21.0%	0.013
**Admission blood glucose (mg/dL)**	408.1 ± 163.8	513.7 ± 268.0	<0.001
**Fructosamine (μmol/L)**	506.4 ± 142.7	649.9 ± 160.6	<0.001
**HbA1c (mmol/mol)**	96.5 ± 22.8	108.0 ± 24.6	0.001
**C-peptide (ng/mL)**	0.58 ± 0.42	0.51 ± 0.37	NS
**Total cholesterol (mg/dL)**	166.3 ± 42.3	210.9 ± 73.3	<0.001
**HDL-cholesterol (mg/dL)**	54.3 ± 28.4	44.9 ± 35.3	<0.001
**IR (IU/kg/day)**	0.80 ± 0.34	1.06 ± 0.37	<0.001
**TSH (microU/mL)**	2.80 ± 2.82	5.51 ± 6.30	0.011
**fT4 (pg/mL)**	10.40 ± 1.18	8.79 ± 2.46	0.038
**Duration of symptoms (days)**	25.5 ± 23.1	27.8 ± 23.6	0.025
**Duration of hydration (hours)**	18.6 ± 36.9	25.9 ± 25.0	<0.001
**Duration of hospitalization (days)**	8.9 ± 3.0	10.0 ± 3.2	0.006
**pH**	7.31 ± 0.13	7.21 ± 0.18	<0.001
**HCO_3_^−^ (mmol/L)**	19.4 ± 6.8	14.8 ± 8.3	<0.001

NS: not significant.

**Table 3:** Multivariable linear regression analysis for IR at T1D onset.

Variable	β (Unstandardized)	Standard Error	β (Unstandardized)	95% CI	*p*-Value
**Triglycerides (mg/dL)**	0.000685	0.000190	0.271	[0.000310; 0.001060]	<0.001
**HbA1c (mmol/mol)**	0.002571	0.001126	0.166	[0.000343; 0.004799]	0.024
**pH**	−0.280	0.198	−0.107	[−0.671; 0.112]	0.160
**BMI z-score**	−0.074	0.021	−0.262	[−0.116; −0.033]	<0.001
**C-peptide (ng/mL)**	−0.206	0.069	−0.224	[−0.343; −0.070]	0.003

**Table 4:** Follow-up at 12 months according to IR at T1D onset.

	All Subjects	IR ≤ 1 IU/kg/Day (at T1D Onset)	IR > 1 IU/kg/Day (at T1D Onset)	*p*-Value
**HbA1c (mmol/mol)**	58.65 ± 14.9	58.5 ± 15.5	59.0 ± 14.4	NS
**Total cholesterol (mg/dL)**	172.6 ± 31.5	173.9 ± 33.7	170.8 ± 27.2	NS
**HDL-cholesterol (mg/dL)**	59.9 ± 13.7	61.3 ± 14.1	57.4 ± 12.9	NS
**Triglycerides (mg/dL)**	67.2 ± 39.9	68.4 ± 65.5	65.1 ± 24.6	NS
**C-peptide (ng/mL)**	0.54 ± 0.63	0.63 ± 0.77	0.43 ± 0.38	NS
**IR (IU/kg/day)**	0.64 ± 0.31	0.60 ± 0.25	0.72 ± 0.38	0.007

NS: not significant.

## Data Availability

The de-identified data presented in this study are available on request from the corresponding author, subject to institutional and data-protection regulations.
